# Small Intestinal Bacterial Overgrowth in Patients With Severe Alcoholic Hepatitis: Frequency and Predictors

**DOI:** 10.7759/cureus.85516

**Published:** 2025-06-07

**Authors:** Venkatesh Vaithiyam, Sanjeev Sachdeva, Payila Aneesh, Ravi Teja Reddy, Rahul Chittem, Aarushi Ahuja, Kartik Mehta, Ashok Dalal, Ajay Kumar

**Affiliations:** 1 Gastroenterology, Govind Ballabh Pant Institute of Postgraduate Medical Education and Research, New Delhi, IND

**Keywords:** alcohol-related liver disease, gastrointestinal motility, gut microbiota dysbiosis, hydrogen breath test, portal hypertension

## Abstract

Background and aims: Small intestinal bacterial overgrowth (SIBO) is a gut microbiota-related disorder characterized by excessive bacterial density and/or abnormal microbiota composition in the small intestine. We aimed to evaluate the frequency of SIBO in patients with severe alcoholic hepatitis (SAH) and to identify independent predictors.

Methods: We included 60 consecutive patients with SAH and 30 healthy controls (HC). After an overnight fast, SIBO was evaluated using the glucose hydrogen breath test (GHBT) with 100 g of glucose. A sustained increase in breath hydrogen or methane levels > 12 parts per million (ppm) above the basal level was considered diagnostic for SIBO.

Results: SIBO was more frequent in patients with SAH than HC (15/60 (25.0%) vs. 1/30 (3.3%), p = 0.017). In the univariate analysis, globulin levels, total protein levels, and predominant methane producer status significantly differed between patients with and without SIBO. In the multivariate analysis, the predominant methane producer status increased the risk of SIBO by 17.44 times (adjusted odds ratio: 17.44 (95% CI (1.9-154.04)), p = 0.001).

Conclusions: This study showed that SIBO is a more common finding in patients with SAH than in HC. The predominant methane producer status independently predicted the SIBO in these patients.

## Introduction

The gut microbiota is a rich and dynamic community of microorganisms, and alterations in either quantity or quality can lead to diseases. The total number of microbes in the gastrointestinal (GI) tract varies and increases in the craniocaudal direction. The total gut microbiota is approximately 10^14^, more than 10 times the total number of human cells [1]. Dysbiosis or altered gut composition is associated with the pathophysiology of numerous inflammatory and infectious disorders [2].

Small intestinal bacterial overgrowth (SIBO) is a dysregulated gut microbiota-related disease characterized by excessive density and/or abnormal microbiota composition in the small bowel. It arises from a disruption of the normal homeostatic systems that regulate intestinal bacterial populations. Risk factors for SIBO include gastric acid suppression, gastric bypass, changes in bile acid metabolism, altered orocecal transit time, Crohn’s disease, chronic pancreatitis, small intestinal diverticula, opiate and alcohol consumption, and liver cirrhosis [3].

SIBO is typically defined as the presence of ≥ 10^5^ colony-forming units (CFU)/mL of bacteria in small intestinal fluid aspirate [4]. The diagnosis of SIBO is challenging, and aspiration and culture of the luminal contents of the small bowel are considered the gold standard tests for detection. However, this procedure is time-consuming, expensive, and invasive; lacks standardization; requires dedicated infrastructure; and can result in sampling errors. Therefore, the most common testing methods for SIBO are the hydrogen (H2) and methane (CH4) breath tests because of their safety, simplicity, lack of invasiveness, and low cost [5].

In 1957, Martini et al. showed that patients with cirrhosis had higher *Enterococcus faecalis* levels in the small intestine than healthy controls [6]. This was the first study to identify alterations in the gut microbiota of patients with liver cirrhosis, and subsequent studies have confirmed the presence of dysbiosis in liver cirrhosis. SIBO has been found in as many as two-thirds of patients with cirrhosis, and its presence has been associated with cirrhosis complications [7,8].

Alcohol consumption is a significant cause of liver cirrhosis and portal hypertension. Severe alcohol-associated hepatitis is associated with bacterial infections and the development of acute-on-chronic liver failure, multiorgan failure, and high short-term mortality ranging from 10% to 50% [9]. Patients with alcoholic hepatitis have elevated portal pressure, regardless of the presence of cirrhosis, which may lead to intestinal dysmotility and SIBO. Alcohol and its metabolites can adversely affect the mucosa of the small intestine, GI motility, and the immune system, and alcoholics are more likely to develop SIBO. Studies by Mutlu et al. and Schnabl et al. demonstrated that alcohol promotes dysbiosis and bacterial overgrowth, and these Gram-negative bacteria release bacterial endotoxins [10,11]. Endotoxins activate proteins and immune cells, promote gut inflammation and liver injury, and may increase GI tract (GIT) inflammation and intestinal hyperpermeability, resulting in endotoxemia and systemic inflammation.

Individual data regarding the influence of alcohol consumption, portal hypertension, and liver cirrhosis on the prevalence of SIBO are available. However, there are no studies on the prevalence of SIBO in alcoholic hepatitis where the combined effects of portal hypertension and alcoholism are present. This study aimed to evaluate the frequency and predictors of SIBO in patients with severe alcoholic hepatitis.

## Materials and methods

We prospectively included 60 consecutive patients with severe alcoholic hepatitis admitted to the Gastroenterology ward of our institution and 30 healthy controls (HC) between May 2021 and January 2023. The patients were aged between 18 and 60 years, with an average alcohol intake of more than 80 g per day for men and 60 g per day for women during the last three months before enrolment, sustained for at least five years. Patients with rapid-onset jaundice in the absence of biliary obstruction, aspartate transaminase (AST)/alanine transaminase (ALT) > 2, Maddrey’s discriminant function ≥ 32, or liver histologic findings (whenever available) consistent with alcoholic hepatitis were included.

Subjects with predisposing conditions for SIBO (e.g., prior intestinal surgery, hypothyroidism, diabetes, and scleroderma) were excluded. Additionally, subjects who had taken subjects who had taken proton pump inhibitors, antibiotics, promotility drugs, or antimotility drugs within the preceding six weeks were excluded. Patients with concomitant hepatorenal syndrome, hepatocellular carcinoma, uncontrolled bacterial infection, spontaneous bacterial peritonitis, upper gastrointestinal bleeding, hepatic encephalopathy, or human immunodeficiency virus (HIV) infection were excluded. Patients with AST and ALT levels > 500 IU/L and jaundice for more than six months were excluded.

Ethical clearance was obtained from the Institutional Ethics Committee (F.1/IEC/MAMC/85/04/2021/No. 469). Written informed consent was obtained from all participants. The demographic data of all cases and controls were recorded. All cases underwent the following investigations: complete haemogram, renal and liver function tests, PT-INR, glucose hydrogen breath test (GHBT), stool microscopy, and workup for alcoholic liver disease. Both cases and controls underwent evaluation for SIBO using a GHBT. Differences in the frequency of SIBO between cases and controls and various predictive parameters of SIBO among alcoholics were evaluated.

SIBO was evaluated using the GHBT. After overnight fasting, the GHBT was performed using 100 g of glucose (dissolved in 200 mL of water). The subjects were instructed to avoid slow-absorbing carbohydrates and fibres during the 48 hours before the test. Any lactulose subject was told to stop it three days before the test. Chewing of tobacco, any form of exercise, and smoking were refrained from two hours before and during the procedure. The subjects were told to brush their teeth and perform a chlorhexidine mouthwash before the commencement of the test. Breath samples were analysed by a QuinTron BreathTracker SC Analyser (QuinTron Instrument Company, Milwaukee, WI) for hydrogen and methane concentrations. Breath methane and hydrogen concentrations were observed at baseline (i.e., before glucose administration), and an average of four values was taken as the basal level. The participants were then instructed to consume 100 g of glucose mixed with 200 mL of water. Subsequently, breath methane and hydrogen were recorded every 15 minutes for three hours. A persistent elevation in breath methane or hydrogen levels of more than 12 parts per million (ppm) above the basal level (at least two readings) indicated SIBO. Subjects with a fasting methane level >10 ppm were labelled as predominant methane producers (PMP)​​​.

Statistical analysis

The sample size was calculated using the Epi-Info (Centers for Disease Control and Prevention, Atlanta, GA) software; the patient control ratio was 2:1, and the power of the study was 80%, with a two-sided confidence interval (CI) of 95%. (Because there were no studies on the prevalence of SIBO among patients with alcoholic hepatitis, the frequency of SIBO in alcoholic liver disease was taken into account. The average prevalence of SIBO among patients with alcoholic liver disease and healthy subjects was 34.05% and 6.65%, respectively, with sample sizes of 52 and 26 patients and controls.) Therefore, we included 60 consecutive patients with severe alcoholic hepatitis and 30 HCs.

Continuous variables were expressed as medians and ranges, whereas categorical variables were expressed as proportions or percentages. Continuous variables were compared between the two groups using the Mann-Whitney U test, whereas categorical variables were compared using Fisher’s exact test. Univariate and multivariate analyses were performed to identify the predictors of SIBO. The Statistical Product and Service Solutions (SPSS, version 21.0; IBM SPSS Statistics for Windows, Armonk, NY) software was used for statistical analysis. A p-value of < 0.05 was considered statistically significant.

## Results

During the study period, 145 patients with severe alcohol consumption and jaundice were enrolled. Among these, 85 were excluded for various reasons. Co-existing viral hepatitis was found in 10 patients, of whom three were positive for IgM hepatitis A virus, five were positive for IgM hepatitis E virus, and two had hepatitis B infection. Twenty-five patients were excluded due to a recent hospital stay or antibiotic intake. Eight patients were diagnosed with spontaneous bacterial peritonitis, and 15 with acute kidney injury. Ten patients could not undergo the GHBT, and 17 had persistently high basal hydrogen levels. After excluding 85 patients, 60 patients were included in the study. We also did a glucose hydrogen breath test for 30 healthy controls.

The median age of the patients with severe alcoholic hepatitis (38 years, range: 28-52) was comparable to that of the controls (38 years, range: 25-59; p = 0.631). All patients and controls were male. The patterns of alcohol intake, complications of severe alcoholic hepatitis, and the severity of alcoholic hepatitis were recorded. A positive GHBT result (i.e., indicative of SIBO) was more frequent in patients than in healthy controls (15/60 (25%) vs. 1/30 (3.33%); p = 0.017) (Table 1).

**Table 1:** Frequency of SIBO among severe alcoholic hepatitis and healthy controls based on the GHBT GHBT – glucose hydrogen breath test, SIBO – small intestinal bacterial overgrowth

S. No	SIBO	Cases (N=60)	Controls (N=30)	p- value
1	Present	15	1	0.017
2	Absent	45	29	

Basal hydrogen and methane levels were comparable between the cases and healthy controls. There was no significant difference between the cases and controls in terms of PMP (8/60 (13.3%) vs. 3/30 (10%); p = 0.746). Peak hydrogen and methane showed significant differences between cases and healthy controls (Table 2).

**Table 2:** Characteristics of the GHBT GHBT – glucose hydrogen breath test

S. No	Parameters	Cases (N=60)	Controls (N=30)	Test Statistic	p-value
1	GHBT – Baseline (ppm) (Median (Range))	4 (0-12)	5 (2-12)	838.0	0.592
2	GHBT – Peak (ppm) (Median (Range))	9 (1-89)	5 (2-21)	662.0	0.041
3	Predominant Methane Producers (n (%))	7 (11.6%)	3 (10%)		0.746
4	Methane – Baseline (ppm) (Median (Range))	5 (0-15)	5 (0-11)	889.5	0.928
5	Methane – Peak (ppm) (Median (Range))	9 (0-29)	5 (1-14)	626.0	0.018
6	Symptoms During GHBT (n (%))	3 (5%)	0%		0.548

On univariate analysis of various variables among SIBO-positive SAH and SIBO-negative SAH (age, symptoms, pattern of alcohol intake, complications of alcoholic hepatitis, clinical parameters, laboratory parameters, and basal breath hydrogen), only three parameters were significantly different, which included serum globulin, total protein more than 7.5 g/dL, and PMP status. Two additional variables showed a trend towards significance, namely, HR > 90 beats per minute and presence of ascites (Table 3).

**Table 3:** Univariate analysis of the predictors of SIBO ALP – alkaline phosphatase, ALT – alanine aminotransferase, AST - aspartate transaminase, BMI – body mass index, CLD – chronic liver disease, cm - centimeter, CTP – Child-Turcotte-Pugh, GHBT – glucose hydrogen breath test, g/dL - gram per deciliter, Hb – hemoglobin, HR – heart rate, INR – international normalized ratio, kg – kilogram, MDF – Maddrey Discriminant Function, MELD – Model for End-Stage Liver Disease, mg/dL – milligrams per deciliter, mEq/L – milliequivalents per liter, min – minute, mm³ – cubic millimeter, mmHg – millimeters of mercury, ppm – parts per million, PT – prothrombin time, SBP – systolic blood pressure, SIBO – small intestinal bacterial overgrowth, WBC – white blood cell count

S. No	Parameters	SIBO Present (N=15)	SIBO Absent (N=45)	Test Statistic	p-value
Demographic parameters					
1	Age (years) (Median (Range))	39 (28-51)	38 (28-52)	350	0.831
Symptoms of SIBO					
2	Abdominal bloating (n(%))	6 (40%)	12 (26.6%)		0.347
3	Flatulence (n(%))	1 (6.7%)	5 (11.1%)		1.000
4	Belching (n(%))	1 (6.7%)	3 (6.7%)		1.000
5	Nausea (n(%))	1 (6.7%)	2 (4.4%)		0.441
6	Vomiting (n(%))	1 (6.7%)	6 (13.3%)		0.668
7	Epigastric pain (n(%))	1 (6.7%)	5 (11.1%)		1.000
8	Incomplete evacuation (n(%))	0 (0%)	1 (2.2%)		1.000
9	Diarrhea (n(%))	2 (13.3%)	3 (6.7%)		0.591
10	Constipation (n(%))	2 (13.3%)	4 (8.9%)		0.634
Patterns of Alcohol Intake					
11	Duration of alcohol intake (months) (Median (Range))	120 (60-240)	132 (60-324)	277.5	0.302
12	Content per day (grams) (Median (Range))	80 (80-360)	80 (60-100)	417	0.164
13	Last intake (days) (Median (Range))	25 (4-90)	30 (7-90)	300	0.514
Complications of Alcoholic Hepatitis					
14	Duration of jaundice (days) (Median (Range))	25 (2-90)	25 (3-90)	328.50	0.877
15	Ascites (n(%))	14 (93.3%)	32 (71.1%)		0.053
16	Hepatic encephalopathy (n(%))	1 (6.7%)	5 (11.1%)		1.000
17	Esophageal or gastric varices (n(%))	4 (26.7%)	16 (35.6%)		0.753
18	Chronic liver disease (n(%))	7 (46.7%)	19 (42.2%)		0.773
Clinical Parameters					
19	Height (cm) (Median (Range))	160 (154-170)	165 (150-176)	279.0	0.310
20	Weight (kgs) (Median (Range))	62 (46-91)	56 (40-91.9)	388	0.388
21	BMI > 25 (n(%))	6 (40%)	8 (17.8%)		0.253
22	Heart Rate > 90/min (n(%))	11 (73.3%)	19 (42.2%)		0.072
23	Systolic blood pressure (mmHg) (Median (Range))	110 (90-140)	109 (80-145)	333	0.938
Laboratory Parameters					
24	Hb (g/dL) (Median (Range))	10 (7-14.2)	10.1 (6.6-13.4)	331	0.912
25	Leukocytosis (n(%))	10 (66.7%)	28 (62.2%)		0.719
26	Neutrophil (%) (Median (Range))	80 (63-91)	85 (50-90)	283	0.346
27	Platelets (×10⁵/mm³) (Median (Range))	1.3 (0.27-3.0)	1.05 (0.11-3.99)	343	0.925
28	Prothrombin time (secs) (Median (Range))	20 (16-31.2)	20 (15-48)	314	0.686
29	INR (Median (Range))	1.7 (1.3-2.7)	1.8 (1.3-5.7)	293.5	0.450
30	Urea (mg/dL) (Median (Range))	20 (3-56)	18 (6-42)	323	0.804
31	Creatinine (mg/dL) (Median (Range))	0.8 (0.3-1.1)	0.7 (0.44-1.2)	334.4	0.959
32	Sodium (mEq/L) (Median (Range))	130 (125-145)	131 (124-140)	318	0.734
33	Potassium (mEq/L) (Median (Range))	4 (3.5-4.9)	4 (2.6-5.4)	391.5	0.333
34	Total Bilirubin (mg/dL) (Median (Range))	7.8 (3-16.8)	10 (2.6-45.2)	301.0	0.533
35	AST (IU/L) (Median (Range))	191 (96-330)	166 (74-337)	372.5	0.550
36	ALT (IU/L) (Median (Range))	55 (21-168)	70 (20-207)	247.5	0.124
37	ALP (IU/L) (Median [Range])	123 (61-208)	110 (61-279)	391.5	0.356
38	Total protein (> 7.5 g/dl) (n(%))	10 (66.6%)	13 (28.9%)		0.014
39	Albumin (g/dL) (Median (Range))	2.9 (2–4)	3 (1.9-4.1)	301	0.531
40	Globulin (g/dL) (Median (Range))	4.9 (3.1-6.4)	4.1 (2.6-6.2)	474.5	0.019
41	MELD (Median (Range))	24 (18-32)	24 (16-39)	268.5	0.237
42	CTP (Median (Range))	11 (8-12)	11 (7-14)	336.5	0.986
43	MDF (Median (Range))	45.3 (32.6–95.9)	48 (32-178.5)	277.5	0.306
Characteristics of GHBT					
44	Basal hydrogen (ppm) (Median (Range))	5 (0–10)	4 (0-12)	567.5	0.794
45	Predominant methane producer status (n(%))	5 (33.3%)	2 (6.7%)		0.008

We performed multivariate analysis for the variables that were significant in the univariate analysis and found that predominant methane producers were significantly associated with an increased risk of SIBO, with an adjusted odds ratio of 17.44 (95% CI: 1.9-154.04; p = 0.01) (Table 4).

**Table 4:** Multivariate analysis of the predictors of SIBO Statistical test: Multinominal logistic regression. CI – confidence interval, SIBO – small intestinal bacterial overgrowth

S. No	Parameters	B	SE	Wald	p-value	Adjusted Odds Ratio (95% CI)
1	Globulin	0.981	0.532	3.519	0.061	2.667 (0.9-7.434)
2	Total protein of more than 7.5 g/dL	0.623	0.832	0.560	0.454	1.8 (0.3-9.513)
3	Predominant methane producers	2.859	1.113	6.597	0.01	17.44 (1.9-154.04)

## Discussion

Autonomic dysfunction, gut dysmotility, and altered gut microbiota have been considered in the pathogenesis of SIBO in patients consuming alcohol. It has been suggested that SIBO plays a role in cirrhotic complications. Various studies have evaluated changes in faecal microbiota among patients with alcoholic hepatitis [12-15], but none have assessed the frequency of SIBO in patients with severe alcoholic hepatitis. Our study showed that SIBO was more frequent in patients with alcoholic hepatitis than healthy individuals (25% vs. 3.3%). The difference in the prevalence of SIBO was statistically significant (p < 0.017). However, the published frequency of SIBO in patients with alcoholism and cirrhosis varies depending on the study population as well as the methods and criteria for diagnosing SIBO.

In a study conducted by Bode et al., the frequency of SIBO in alcoholic liver disease was 37.8%, which was slightly higher than that observed in our study [16]. However, 13.3% of the healthy control population had SIBO, which was much higher than that in our control group. Historically, the frequency of SIBO in healthy controls ranged from 0% to 20 % [17,18]. Bode et al. also reported a noteworthy difference in the prevalence of SIBO between patients with and without alcoholic cirrhosis (42% vs. 33.3%) [16]. Gabbard et al. showed that the frequency of SIBO in patients with alcoholism (but without cirrhosis) was 58 %, which is much higher than that in our study [19]. They calculated the frequency of SIBO using the lactose hydrogen breath test. The GHBT is preferred over the LHBT for the diagnosis of SIBO, as the GHBT has higher sensitivity, and the same was used in our study [19].

Maslennikov et al., in a systematic review, reported the frequency of SIBO among patients with cirrhosis as 40.7%, which was higher than that observed in the present study [20]. This study also noted heterogeneity in the detection of SIBO, which further emphasises the need for universally standardised diagnostic criteria for SIBO. In their study, there was no difference in the prevalence of SIBO among various etiologies. Still, they showed a linear relationship between the occurrence of SIBO and the severity of liver disease [20].

In our study, there was no difference between basal hydrogen breath levels among patients and controls (median: 4 vs. 5; p = 0.794), whereas a study by Gerova et al. showed a substantial difference in basal hydrogen levels in subjects with cirrhosis compared to healthy controls (15.54 vs. 6.24; p < 0.05) [21]. Among the multivariate analyses, only the predominant methane producer status at baseline was independently associated with SIBO, contributing to a 17-fold higher risk of SIBO in patients with SAH. This association may indicate altered gut microbiota in subjects with SAH at baseline. It has been shown that methane-producing bacteria, such as Methanobrevibacter smithii, are often more resistant to antibiotics [22]; therefore, identification of methane-producing bacteria in the small bowel is essential. Yokoyama et al. studied the role of hydrogen-producing small intestinal bacterial overgrowth (H-SIBO) in hepatic encephalopathy and liver function. They showed that the prevalence of methanogenic SIBO (M-SIBO) was 14% and H-SIBO was 14.9% in their study population [22]. Yokoyama et al. also showed that the therapeutic response to rifaximin was lower in M-SIBO than in H-SIBO (20% vs. 67%) [22].

Among the various baseline parameters, serum globulin and total protein levels were significantly associated with SIBO. At the same time, the presence of ascites and heart rate > 90 beats/min showed positive trends with SIBO but did not reach a significance value of p < 0.05. Alcoholic hepatitis is a form of profound systemic inflammation, and hyperglobulinemia is commonly observed in patients with inflammation. Liu et al. showed that hyperimmunoglobulinemia and high plasma LPS levels were observed in both patients with chronic liver diseases and animal models [23]. They also found that hyperimmunoglobulinemia in chronic liver disease arises mainly from collateral circulation secondary to portal hypertension, gut antigens, and endotoxins that bypass the liver and reach the antibody-producing cells [23].

The amount of alcohol consumed and the time of the last alcohol intake were not associated with SIBO in our study. In contrast, Gabbard et al. showed that moderate alcohol consumption is associated with SIBO, but they did not study the duration of alcohol intake in their study [19]. Our study did not observe this relationship, possibly due to the modest sample size and exclusion of sicker patients, as they could not undergo GHBT.

Morencos et al. reported SIBO to be more frequent in patients with ascites compared to patients without ascites (37.1% vs. 5.3%), which was not observed in our current study (93.3% vs. 71.1%; p = 0.053) [24]. Pande et al. found that a blend of ascites and a serum bilirubin level ≥ 2 mg/dL had an 82% increased risk of SIBO. In contrast, the presence of only ascites had a 63% increased chance of SIBO, whereas patients with neither had a 10% chance of developing SIBO [25]. In our study, serum bilirubin was not significant, while ascites showed a trend towards significance. Studies have shown that SIBO is more commonly observed in patients with SBP than in those without SBP [26,27]. However, we excluded patients with SBP from our study to prevent the confounding effect of SBP on systemic inflammation in patients with SAH.

Pande et al. reported that SIBO was more frequent in patients with decompensated cirrhosis and higher in CTP C than in CTP A (73% vs. 20%) [24]. This finding was also observed by Pande et al., who showed that SIBO was higher in CTP-C than in CTP-B (48.3% vs. 13.4%) [25]. However, this association of disease severity based on the CTP score was not observed in our study; other clinical liver severity parameters, such as MELD and mDF, were not significantly associated with SIBO in our study. This non-association between SIBO and clinical severity was also observed in the study by Gerova et al., who showed that SIBO was common in patients with liver cirrhosis but was not associated with severity [21].

The severity parameters of liver disease are significant predictors of SIBO occurrence, as shown in previously published data. However, few studies, such as ours, have demonstrated that liver disease severity is not a risk factor for SIBO prevalence. The likely explanation may be the small sample size and limited sensitivity of the GHBT to uncover/diagnose SIBO. Ghoshal et al. showed that the sensitivity of the GHBT was 27%, but the specificity was 100% [28], which is one of the study's limitations. Hence, further studies with larger sample sizes and multicenter designs should be conducted using multiple methods such as breath tests and jejunal aspirate culture [29]. Moreover, newer tests, such as the 16S ribosomal RNA sequencing analysis from jejunal aspirates, may be more effective than GHBT and culture in detecting SIBO.

## Conclusions

The present study revealed that SIBO was more frequent in patients with alcoholic hepatitis than in healthy controls. The presence of predominant methane producers was found to be an independent risk factor for SIBO in patients with SAH. Hyperglobulinemia is a manifestation of inflammation, and high globulin levels in our study were associated with SIBO in patients with SAH. Although our study did not find a relationship between the duration or quantity of alcohol intake, liver disease severity scores, and the occurrence of SIBO, further studies with larger sample sizes, multicentre designs, and the use of more sensitive and specific tests for SIBO may identify additional predictors of SIBO in severe alcoholic hepatitis. Our study is the first to evaluate SIBO in SAH. It should encourage further research in this field, particularly concerning the impact of SIBO treatment in SAH, which otherwise has poor outcomes.
